# Effects of Semaglutide on Stroke Subtypes in Type 2 Diabetes: Post Hoc Analysis of the Randomized SUSTAIN 6 and PIONEER 6

**DOI:** 10.1161/STROKEAHA.121.037775

**Published:** 2022-05-18

**Authors:** W. David Strain, Ofir Frenkel, Martin A. James, Lawrence A. Leiter, Søren Rasmussen, Peter M. Rothwell, Maria Sejersten Ripa, Thomas C. Truelsen, Mansoor Husain

**Affiliations:** University of Exeter Medical School, St Luke’s Campus, United Kingdom (W.D.S., M.A.J.).; Academic Department of Healthcare for Older People, Royal Devon and Exeter NHS Foundation Trust, United Kingdom (W.D.S., M.A.J.).; Novo Nordisk A/S, Søborg, Denmark (O.F., S.R., M.S.R.).; Li Ka Shing Knowledge Institute, St Michael’s Hospital (L.A.L.), University of Toronto, ON, Canada.; Ted Rogers Centre for Heart Research (M.H.), University of Toronto, ON, Canada.; Wolfson Centre for Prevention of Stroke and Dementia, Nuffield Department of Clinical Neurosciences, John Radcliffe Hospital, University of Oxford, United Kingdom (P.M.R.).; Department of Neurology, University of Copenhagen, Rigshospitalet, Denmark (T.C.T.).

**Keywords:** atrial fibrillation, blood pressure, myocardial infarction, peptides, prevalence

## Abstract

GLP-1 RA (glucagon-like peptide-1 receptor agonists), including semaglutide, may reduce stroke risk in people with type 2 diabetes. This post hoc analysis examined the subcutaneous and oral semaglutide effects, versus placebo, on stroke and its subtypes in people with type 2 diabetes at high cardiovascular risk.

**Methods:**

SUSTAIN 6 (Trial to Evaluate Cardiovascular and Other Long-Term Outcomes With Semaglutide in Subjects With Type 2 Diabetes) and PIONEER 6 (Peptide Innovation for Early Diabetes Treatment) were randomized cardiovascular outcome trials of subcutaneous and oral semaglutide in people with type 2 diabetes at high cardiovascular risk, respectively. Time to first stroke and stroke subtypes were analyzed using a Cox proportional hazards model stratified by trial with pooled treatment as a factor. The impact of prior stroke, prior myocardial infarction or stroke, age, sex, systolic blood pressure, estimated glomerular filtration rate, and prior atrial fibrillation on treatment effects was assessed using interaction *P* values. Risk of major adverse cardiovascular event was analyzed according to prior stroke.

**Results:**

A total of 106/6480 participants had a stroke (1.0 event/100 patient-years of observation [PYO]). Semaglutide reduced incidence of any stroke versus placebo (0.8 versus 1.1 events/100 PYO; hazard ratio, 0.68 [95% CI, 0.46–1.00]; *P*=0.048), driven by significant reductions in risk of small-vessel occlusion (0.3 versus 0.7 events/100 PYO; hazard ratio, 0.51 [95% CI, 0.29–0.89]; *P*=0.017). Hazard ratios for risk of any stroke with semaglutide versus placebo were 0.60 (95% CI, 0.37–0.99; 0.5 versus 0.9 events/100 PYO) and 0.89 (95% CI, 0.47–1.69; 2.7 versus 3.0 events/100 PYO) in those without and with prior stroke, respectively. Except for prior atrial fibrillation (*P*_*interaction*_=0.025), no significant interactions were observed between treatment effects on risk of any stroke and subgroups investigated, or between treatment effects on risk of major adverse cardiovascular event and prior stroke (*P*_interaction_ >0.05 for all).

**Conclusions:**

Semaglutide reduced incidence of any first stroke during the trials versus placebo in people with type 2 diabetes at high cardiovascular risk, primarily driven by small-vessel occlusion prevention. Semaglutide treatment, versus placebo, lowered the risk of stroke irrespective of prior stroke at baseline.

**Registration:**

URL: https://www.clinicaltrials.gov; Unique identifier: NCT01720446 and NCT02692716.

As the prevalence of diabetes is rapidly increasing, the number of people with complications, including stroke, is expected to increase.^1^ Epidemiological studies show that diabetes increases the risk of stroke by ≈2-fold.^2^ The prevalence of stroke was 18.6% in people with type 2 diabetes (T2D) in 2015.^3^ People with diabetes are more likely to have a stroke at a younger age, with worse outcomes and higher risk for recurrence compared with those without diabetes.^4,5^ The 10-year recurrence rate of stroke was reported to be 11.2% in people who had a stroke,^6^ and those with diabetes had 45% to 60% higher risk of stroke recurrence than those without diabetes.^7,8^ This suggests that diabetes is an independent risk factor for stroke recurrence.^8^ Among the subtypes of strokes, cerebral small vessel disease is more common in people with T2D, compared with those without T2D. Thus, prevention of stroke, including all its subtypes, should be a major concern in the clinical management of people with diabetes.

Accumulating evidence suggests that GLP-1 RAs (glucagon-like peptide-1 receptor agonists) may reduce the risk of stroke beyond their glycemic impact in people with T2D.^9–14^ Currently, there are 5 available subcutaneous (SC) GLP-1 RAs (exenatide, lixisenatide, liraglutide, dulaglutide, and semaglutide)^15,16^ and one oral formulation (once-daily oral semaglutide).^15^ In a large meta-analysis of recent cardiovascular outcome trials, GLP-1 RAs were reported to reduce the risk of stroke significantly in people with T2D compared with placebo (hazard ratio [HR], 0.83 [95% CI, 0.76–0.92]; *P*=0.0002).^14^ The American Heart Association/American Stroke Association as well as endocrinology and cardiology guidelines recommend the use of GLP-1 RAs with evidence of cardiovascular benefit for individuals at high/very high cardiovascular risk regardless of glycemic control to reduce the risk of future vascular events.^7,17,18^ Despite the short duration of the trials, semaglutide has demonstrated a consistent reduction in major adverse cardiovascular events (MACEs) in the SUSTAIN 6 (Trial to Evaluate Cardiovascular and Other Long-Term Outcomes With Semaglutide in Subjects With Type 2 Diabetes), with once-weekly SC semaglutide,^19^ and the PIONEER (Peptide Innovation for Early Diabetes Treatment) 6 trial, with once-daily oral semaglutide.^20^

The present post hoc analysis, therefore, aimed to examine the effect of semaglutide, irrespective of whether administered subcutaneously or orally, on stroke risk, stratified by subtype, in people with T2D at high cardiovascular risk, using pooled data from the SUSTAIN 6 and PIONEER 6 trials.

## Methods

De-identified individual participant data and redacted Clinical Study Report will be available according to Novo Nordisk data sharing commitments.

### Trial Overview

SUSTAIN 6 (NCT01720446) and PIONEER 6 (NCT02692716) were global, randomized, phase 3 trials assessing the cardiovascular safety of once-weekly SC and once-daily oral semaglutide versus placebo, respectively.^19,20^ People with T2D aged ≥50 years with established cardiovascular disease (myocardial infarction, history of symptomatic coronary heart disease, coronary, carotid or peripheral arterial revascularization, stroke, transient ischemic attacks, >50% stenosis of coronary, carotid or lower extremities arteries), chronic heart failure or chronic kidney disease, or ≥60 years with cardiovascular risk factors were included in both trials.^19,20^ Stroke was defined as an acute episode of neurological dysfunction caused by focal or global brain, spinal cord, or retinal vascular injury, and transient ischemic attacks (as used in the inclusion criteria of both trials) was defined as a transient (<24 hours) episode of neurological dysfunction caused by focal brain, spinal cord, or retinal ischemia, without acute infarction.^19^ Fatal and nonfatal strokes were adjudicated by an independent adjudication committee.^19,20^ Detailed protocols and trial-specific flow diagrams can be found in the primary SUSTAIN 6 and PIONEER 6 publications.^19,20^

Both SUSTAIN 6 and PIONEER 6 were approved by ethics committees and institutional review boards and were conducted according to the principles of the Declaration of Helsinki. All participants provided written informed consent before participation in trial-related activities.^19,20^

### Post Hoc Analysis

In this exploratory post hoc analysis, the effects of semaglutide versus placebo on time to first occurrence of any type of stroke (fatal and nonfatal; transient ischemic attacks were not included) and stroke subtypes, including ischemic and hemorrhagic strokes, as well as unknown subtypes‚ were investigated. Based on the Trial of ORG 10172 in Acute Stroke Treatment criteria,^21^ ischemic strokes (positively adjudicated events) were further sub-classified into (1) large artery atherosclerosis, (2) cardioembolism, (3) small-vessel occlusion, (4) stroke of other determined cause, and (5) stroke of undetermined cause, by an external blinded stroke physician. In a subgroup analysis, effects of treatment on time to occurrence of any stroke were determined in relation to prior stroke (yes/no), prior myocardial infarction or stroke (yes or no), prior atrial fibrillation (AF; yes or no), age (<75 or ≥75 years), sex (female or male), systolic blood pressure (SBP; <120, ≥120 and ≤140, or >140 mm Hg), and estimated glomerular filtration rate (<60 or ≥60 mL/min/1.73 m^2^). Some of these subgroups were related to the prespecified subgroup analyses for time to first MACE in the primary publications^19,20^ while the others, including prior stroke, prior AF‚ and SBP, were chosen as they were relevant end points related to stroke. The impact of prior stroke on the effect of semaglutide versus placebo on subsequent MACE within the trials was also analyzed. Furthermore, a dose-response analysis was performed to evaluate the impact of individual doses (0.5 mg SC, 1.0 mg SC, and 14 mg oral) of semaglutide versus placebo on time to first occurrence of any stroke. A CONSORT checklist of the current post hoc analysis has been included in the Supplemental Material.

### Statistical Analysis

Data from SUSTAIN 6 and PIONEER 6 were pooled in this analysis, except for the dose-response analysis, where data for individual doses of semaglutide were used, as well as the Aalen-Johansen plots when they were analyzed for individual SUSTAIN 6 and PIONEER 6 trials.

The Aalen-Johansen estimator, a nonparametric estimator of cumulative incidence, was used to calculate the cumulative incidence rates for time to first stroke during the trials, hereby adjusting for all-cause death as a competing risk.^22^ This analysis was performed with data from SUSTAIN 6 and PIONEER 6, pooled and individually. A Cox proportional hazards model stratified by trial with treatment (pooled semaglutide) as a factor was used to examine treatment effects for time to first occurrence of an event. A trial-specific analysis was also performed to investigate whether this treatment effect was homogenous across SUSTAIN 6 and PIOPNEER 6. For the subgroup analyses, subgroups were added as a categorical fixed factor and as an interaction term with treatment. Potential heterogeneity of treatment effect across the subgroups according to prior stroke, prior myocardial infarction or stroke, prior AF, age, sex, SBP, and estimated glomerular filtration rate on time to first occurrence of any stroke during the trials was assessed using interaction *P* values. All analyses were performed using Statistical Analysis System version 9.4 (SAS/STAT 15.1). A *P* value <0.05 was considered as statistically significant. No adjustment for multiple testing was performed, and no direction of any subgroup-treatment effect interaction had been prespecified.

### Role of the Funding Source

The sponsor participated in the design and management of this post hoc analysis, analysis, and interpretation of data. Three of the authors of this article are employees of the sponsor and, as such, were involved in the preparation, review, and approval of the article. All authors had full access to all the data in the analysis and had final responsibility for the decision to submit for publication.

## Results

### Baseline Characteristics

In total, SUSTAIN 6 and PIONEER 6 included 6480 (semaglutide, n=3239; placebo, n=3241) participants, of whom 106 had a stroke and 6374 did not have a stroke during the trials. The mean age of people (±SD) without stroke was 65.4±7.3 years, with the majority of them being male (64.5%), and the mean duration of diabetes was 14.4±8.3 years (Table). The baseline characteristics of people with stroke during the trials treated with semaglutide (n=43) were similar to those treated with placebo (n=63; Table). However, prior stroke (39.5% versus 33.3%) and established cardiovascular disease (93.0% versus 84.1%) were higher in the semaglutide group compared with the placebo group among these individuals (Table).

**Table.**
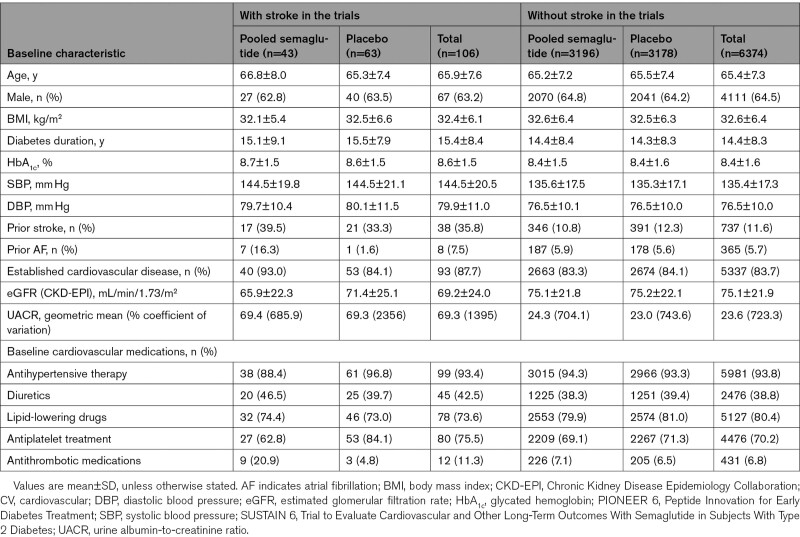
Baseline Characteristics and CV Medications for People With and Without Prior Stroke, Based on Data From the SUSTAIN 6 and PIONEER 6 Trials

### Time to First Stroke and Stroke Subtypes

**Figure 1.**
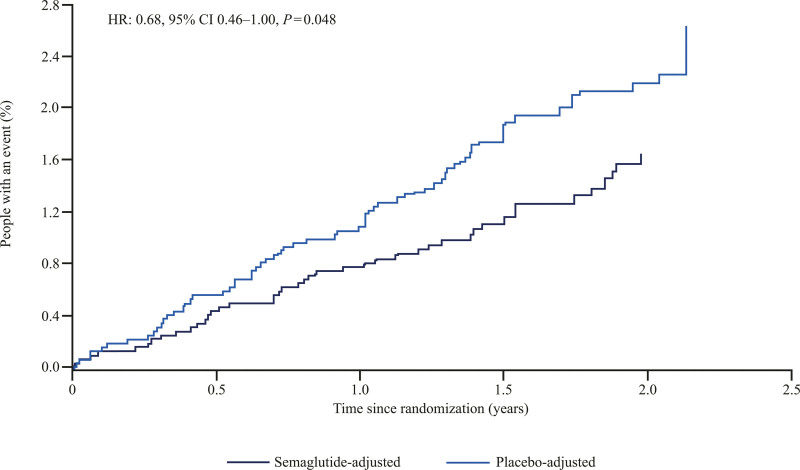
Aalen-Johansen plots for time to first occurrence of any stroke* with the pooled semaglutide versus placebo in people with type 2 diabetes (T2D) at high cardiovascular (CV) risk, based on pooled data from the SUSTAIN 6 (Trial to Evaluate Cardiovascular and Other Long-Term Outcomes With Semaglutide in Subjects With Type 2 Diabetes) and PIONEER 6 (Peptide Innovation for Early Diabetes Treatment) trials. *Included fatal and nonfatal strokes. The cumulative incidence rates for time to first stroke were calculated using Aalen-Johansen method, adjusting for all-cause death as a competing risk. The hazard ratio was estimated from a Cox regression model stratified by trial with treatment (pooled semaglutide vs placebo) as a factor. HR indicates hazard ratio.

**Figure 2.**
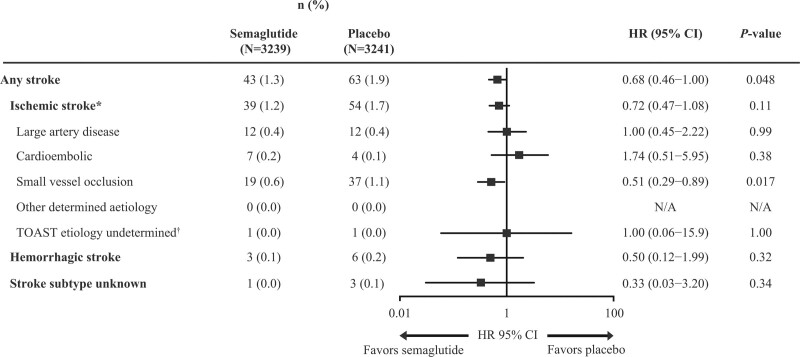
**Effect of semaglutide versus placebo on risk of any stroke and stroke subtypes in people with type 2 diabetes (T2D) at high cardiovascular (CV) risk, based on pooled data from the SUSTAIN 6 (Trial to Evaluate Cardiovascular and Other Long-Term Outcomes With Semaglutide in Subjects With Type 2 Diabetes) and PIONEER 6 (Peptide Innovation for Early Diabetes Treatment) trials.** Data for any stroke and those subtypes based on the TOAST (Trial of ORG 10172 in Acute Stroke Treatment) criteria have been included (transient ischemic attacks were not included in this analysis). Any stroke (including fatal and nonfatal strokes) and the 3 main subtypes were confirmed by the trial-specific Event Adjudication Committee. A Cox proportional hazards model stratified by trial with treatment (pooled semaglutide vs placebo) as a factor was used to examine treatment effects. The trial-specific analysis showed that the treatment effects were homogenous across SUSTAIN 6 (hazard ratio [HR], 0.65 [95% CI, 0.41–1.03]) and PIONEER 6 (HR, 0.76 [95% CI, 0.37–1.56]), *P*_interaction_=0.73. *Ischemic strokes were subcategorized according to TOAST criteria,^21^ by an external, blinded reviewer. †Included patients with ≥2 causes of stroke, undetermined cause despite extensive evaluation, and cause of stroke not known due to cursory evaluation. n indicates number of patients with specified stroke type; N, number of patients in the treatment group; and N/A, not applicable.

The incident rate of any first stroke during the trials was 1.0 event/100 patient-years of observation [PYO]). Administration of semaglutide reduced the risk of any first stroke in the trials compared with placebo (incidence rate: 0.8 versus 1.1 events per 100 PYO; HR, 0.68 [95% CI, 0.46–1.00]; *P*=0.048; Figure 1), with no difference in effect between stroke subtypes: ischemic stroke (incidence rate: 0.7 versus 1.0 events per 100 PYO; HR, 0.72, [95% CI, 0.47–1.08]; *P*=0.11) and hemorrhagic stroke (incidence rate: 0.1 versus 0.1 events per 100 PYO; HR, 0.50 [95% CI, 0.12–1.99]; *P*=0.32; Figure 2).

Among the Trial of ORG 10172 in Acute Stroke Treatment subcategories, small vessel occlusion was the only subcategory to demonstrate a significant treatment difference with semaglutide versus placebo (incidence rate: 0.3 versus 0.7 events per 100 PYO; HR, 0.51 [95% CI, 0.29–0.89]; *P*=0.017; Figure 2).

The effects on risk of any first stroke in the trials with semaglutide versus placebo were consistent in SUSTAIN 6 (incidence rate: 0.9 versus 1.4 events per 100 PYO; HR, 0.65 [95% CI, 0.41–1.03]; *P*=0.07; Figure S1) and PIONEER 6 (incidence rate: 0.6 versus 0.8 events per 100 PYO; HR, 0.76 [95% CI, 0.37–1.56]; *P*=0.45; Figure S2). These were also consistent across the doses used in SUSTAIN 6 (semaglutide 1.0 mg SC versus placebo, incidence rate: 0.8 versus 1.4 events per 100 PYO; HR, 0.56 [95% CI, 0.30–1.03]; *P=*0.06, and semaglutide 0.5 mg SC versus placebo, incidence rate: 1.0 versus 1.4 events per 100 PYO; HR, 0.74 [95% CI, 0.43–1.30]; *P=*0.30).

### Subgroup Analysis

**Figure 3.**
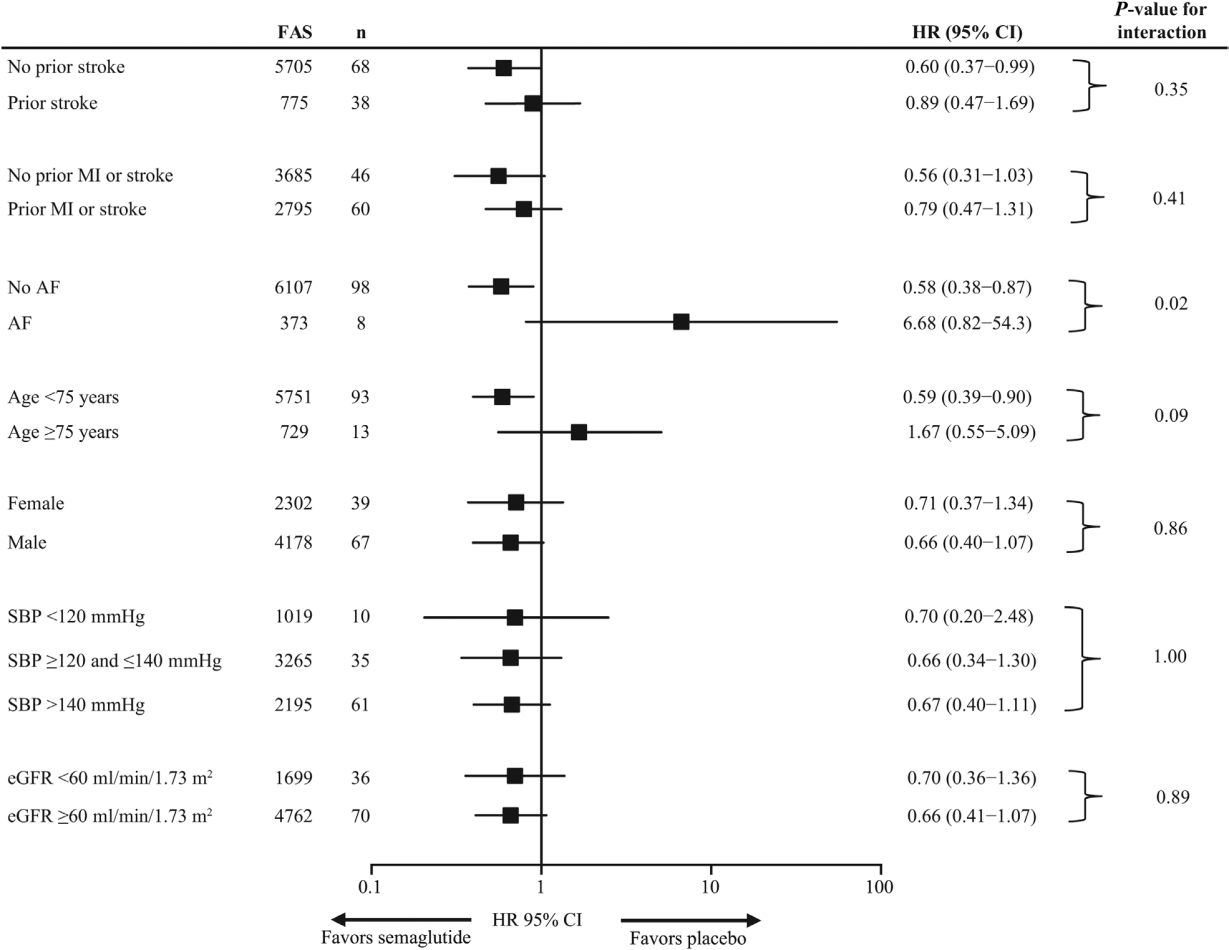
**Effect of semaglutide versus placebo on risk of any stroke stratified by prior stroke, prior myocardial infarction (MI) or stroke, age, sex, systolic blood pressure (SBP), and estimated glomerular filtration rate (eGFR) in people with type 2 diabetes (T2D) at high cardiovascular (CV) risk, based on pooled data from the (Trial to Evaluate Cardiovascular and Other Long-Term Outcomes With Semaglutide in Subjects With Type 2 Diabetes) and PIONEER 6 (Peptide Innovation for Early Diabetes Treatment) trials.** A Cox proportional hazards model stratified by trial with treatment (pooled semaglutide vs placebo) by subgroups as a fixed factor was used to examine treatment effects across the subgroups. AF indicates atrial fibrillation; and HR, hazard ratio.

Data were analyzed using several different baseline characteristics (such as prior stroke [yes/no], prior myocardial infarction or stroke [yes or no], prior AF [yes or no], age [<75 or ≥75 years], sex [female or male], SBP [<120, ≥120 and ≤140, or >140 mm Hg], and estimated glomerular filtration rate [<60 or ≥60 mL/min/1.73 m^2^]). In the subgroup analysis, the risk of any stroke was generally lower in the semaglutide group compared with placebo, except for those with prior AF and those aged ≥75 years (Figure 3). Of note, the incidence of any stroke was nominally reduced in the semaglutide group without history of prior stroke (incidence rate: 0.5 versus 0.9 events per 100 PYO; HR, 0.60 [95% CI, 0.37–0.99]) compared with placebo (Figure 3). However, there were no significant interactions between the treatment effects on the risk of any stroke and the investigated subgroups, except for prior AF (*P*_interaction_=0.025), although the number of events was very low in the prior AF group (7 versus 1 event; Figure 3).

### Time to First MACE

There were no significant interactions between treatment effects on risk of MACE and the prior stroke subgroups (*P*_interaction_=0.56). With semaglutide versus placebo, the incidence rate of MACE was significantly lower among people without prior stroke (2.8 versus 3.8 events per 100 PYO; HR, 0.74 [95% CI, 0.59–0.92]; *P*=0.007) and numerically lower in those with prior stroke (5.2 versus 6.0 events per 100 PYO; HR, 0.86 [95% CI, 0.54–1.36]; *P=*0.52).

## Discussion

In this post hoc analysis of pooled data from the SUSTAIN 6 and PIONEER 6 trials, semaglutide treatment reduced the risk of any stroke in people with T2D at high cardiovascular risk compared with placebo. Furthermore, semaglutide reduced the risk of MACE regardless of prior stroke. Overall, semaglutide showed an encouraging trend for stroke prevention regardless of stroke history and other baseline characteristics.

Recent studies have also suggested a beneficial role of GLP-1 RAs in the prevention of stroke, MACE, and cardiovascular mortality.^23^ Consistent with the current post hoc analysis, a recent systematic review that evaluated the composite outcomes of MACE reported that GLP-1 RAs were associated with significant reduction in the relative risk of stroke (as an individual component of MACE; HR, 0.86 [95% CI, 0.77–0.97]; *P*=0.012).^24^ Of note, in the current study, the significant stroke benefit was driven by a significant reduction in risk of small vessel occlusion with semaglutide versus placebo.

Data analyzed from the REWIND (Researching Cardiovascular Events With a Weekly Incretin in Diabetes) trial showed that dulaglutide reduced the risk of ischemic stroke (HR, 0.75 [CI, 0.59–0.94]; *P*=0.012) but not hemorrhagic stroke (HR, 1.05 [CI, 0.55–1.99]; *P*=0.89), with no effect on stroke severity.^25^ However, in the current post hoc analysis, although semaglutide reduced the risk of any stroke, there were too few hemorrhagic stroke outcomes to determine any specific effect.

The effects on risk of any stroke with semaglutide versus placebo were consistent in SUSTAIN 6 and PIONEER 6, and across the doses used in these trials (weekly 0.5 mg [SC], 1.0 mg [SC], or daily 14 mg [oral]). The pharmacokinetic profiles of SC and oral semaglutide are comparable, with similar exposure-response relationships noted for efficacy and tolerability irrespective of the route of administration.^19,20^ The outcomes noted in SUSTAIN 6 and PIONEER 6 support the use of either route due to the similar clinical benefits and cardiovascular effects following oral or SC administration.^19,20^

The interaction between the effects of semaglutide on participants with and without AF is an interesting observation, given that previous cardiovascular outcome trials have reported no differences in the AF incidence in people receiving GLP-1 RAs compared with placebo.^26^ This finding must be interpreted with caution given that this was based on only 8 events in those with a past history of AF.

There are several potential mechanisms for how GLP-1 RAs may contribute to reduce the risk of stroke in people with T2D. Although lowering of HbA_1c_ by GLP-1 RAs may mediate their effect on cardiovascular outcomes,^27^ GLP-1 RAs can deliver a positive impact on risk of stroke directly, as they exert beneficial effects on smooth muscle cell, endothelial cell, and immune cell function through GLP-1 receptor-dependent and -independent pathways.^23^ Given the speed of divergence of the curves, it is unlikely that the pleiotropic effects of GLP-1 RAs, including reduction of SBP (by 2 to 3 mm Hg) and low-density lipoprotein cholesterol (by −0.1 to −0.2 mmol/L), along with significant weight loss,^28^ would be responsible for the benefit. Treatment with GLP-1 RAs is also considered to exert a neuroprotective effect and may reduce the risk of ischemic strokes.^29^ A recent systematic review has also reported the effects of GLP-1 RAs in reducing ischemic-reperfusion injury in animal models of acute ischemic stroke.^30^ GLP-1 RAs improve neuronal survival, reducing infarct size by up to 75% in rat models of transient middle cerebral artery occlusion.^30^ GLP-1 RAs also generally increase microvascular recruitment and cerebral blood flow, and reduce inflammation, vascular smooth muscle proliferation, oxidative stress‚ carotid intimal-media thickness and other markers of atherosclerosis, thereby affecting both small and large blood vessels.^30,31^ While these mechanisms are likely to explain the impact of semaglutide on small vessel strokes, it is possible that there were too few events of large artery disease to allow any significant effects to be measured.

The safety of semaglutide has been studied in a number of phase 3 trials—semaglutide was associated with fewer serious adverse events versus placebo, although more patients discontinued semaglutide due to adverse events, mainly gastrointestinal.^32,33^

This present study had several limitations. First, the study was an exploratory post hoc analysis, and some baseline characteristics were not protected by the trial randomization, resulting in heterogeneous subgroups. This type of analysis is only hypothesis-forming, and those hypotheses will need to be examined in dedicated trials. Second, due to the low number of people within the subgroups of interest and limited number of events, there is limited statistical power to determine the effects of semaglutide. Further studies with larger patient populations are necessary to establish the specific effects of semaglutide, such as any differential effects in the primary and secondary prevention of stroke‚ and examine our findings conclusively.

## Conclusions

The present post hoc analysis revealed that semaglutide reduced the risk of any stroke compared with placebo in people with T2D at high cardiovascular risk, an effect that appeared to be mediated mainly through a significant reduction in risk of small vessel occlusion. Compared with placebo, treatment with semaglutide lowered the risk of stroke irrespective of prior stroke.

## Article Information

### Acknowledgments

We thank all trial personnel and participants. We contributed significantly to the design and conduct of the study and acquisition of clinical data. Drs Strain and Rasmussen verified the underlying data, and Dr Rasmussen performed the statistical analyses. All authors reviewed and interpreted the data and were involved in drafting and critically revising the article. Ellen Margo Hengeveld, of Novo Nordisk, also kindly reviewed the article. The corresponding author had final responsibility for the decision to submit for publication. All authors approved the final version of the article and take full responsibility for the content. De-identified individual participant data and redacted Clinical Study Report will be available according to Novo Nordisk data sharing commitments. The study protocols of the SUSTAIN 6 (Trial to Evaluate Cardiovascular and Other Long-Term Outcomes With Semaglutide in Subjects With Type 2 Diabetes) and PIONEER 6 (Peptide Innovation for Early Diabetes Treatment) trials are published with the primary publications. The data are available permanently after research completion, and approval of product and product use in both the EU and United States. Data will be shared with bona fide researchers submitting a research proposal requesting access to data and for use as approved by the Independent Review Board according to the IRB Charter (see novonordisk-trials.com). Access request proposal form and access criteria can be found at novonordisk-trials.com. The data will be made available on a specialized SAS data platform.

### Sources of Funding

This study was funded by Novo Nordisk A/S (Søborg, Denmark). Writing and editorial assistance was provided by Jin Heppell, PhD, and Izabel James‚ MBBS‚ of Ashfield MedComms, funded by Novo Nordisk A/S (Søborg, Denmark).

### Disclosures

Dr Strain has received speaker honoraria, conference sponsorship, and unrestricted educational grants from, and/or attended meetings sponsored by, AstraZeneca, Bayer, Eli Lilly, Janssen, Merck, Napp, Novartis, Novo Nordisk, Sanofi Aventis, and Takeda. Dr Strain is supported by the National Institute for Health and Care Research (NIHR) Exeter Clinical Research Facility. The views expressed in this publication are those of the authors and not necessarily those of the NIHR Exeter Clinical Research Facility, the NHS, the NIHR or the Department of Health in England. Dr Frenkel is a Novo Nordisk employee and stockholder. Previous employment at Roche Pharmaceuticals. Dr James has received honoraria and support for conference expenses from Daiichi-Sankyo, Amgen, Portola, and Medtronic. Dr Leiter has received honoraria for advisory board participation from, and has provided continuing medical education on behalf of, AstraZeneca, Bayer, Boehringer Ingelheim, Eli Lilly, Janssen, Merck, Novo Nordisk, Pfizer, Sanofi, and Servier; and has received research grants from AstraZeneca, Bayer, Boehringer Ingelheim, Eli Lilly, Lexicon, Novo Nordisk, and Sanofi. Dr Rasmussen is a Novo Nordisk employee and stockholder. Dr Rothwell has received honoraria for advisory board or trial committee participation from Abbott, Bayer, and Bristol Myers Squibb. Dr Ripa is a Novo Nordisk employee and stockholder. Dr Truelsen has received honoraria for advisory board participation from Boehringer Ingelheim, Medtronic, and Novo Nordisk; speaker honoraria from Boehringer Ingelheim, Medtronic, Bristol Myers Squibb, and AstraZeneca; research grants from Novo Nordisk Foundation; and consultancy fees from Janssen Biotech. Dr Husain has received personal fees from Boehringer Ingelheim and Janssen Inc. for advisory panel consultancy and speaker honoraria; grants and personal fees from AstraZeneca and Merck & Co for advisory panel consultancy and investigator-initiated clinical and preclinical research; personal fees from Roche for advisory panel consultancy; and grants and personal fees from Novo Nordisk for advisory panel consultancy, speaker honoraria, and investigator-initiated preclinical research. Provisional patent for methods for inhibiting platelet aggregation using GLP-1 (glucagon-like peptide-1) receptor agonists, patent No. US61/721,819; patent issued for peptides and methods for preventing ischemic tissue injury, patent No. US61/719,075.

### Supplemental Material

CONSORT checklist

Figures S1–S2

## Supplementary Material


