# Supplementary material for: Effects of Semaglutide on Stroke Subtypes in Type 2 Diabetes: Post Hoc Analysis of the Randomized SUSTAIN 6 and PIONEER 6
Source: Stroke. 2022 May 18;53(9):2749–57. doi: 10.1161/STROKEAHA.121.037775 (PMC9389936; doi:10.1161/STROKEAHA.121.037775)
Supplement: Supplementary file 1 [file str-53-2749-s001.pdf]

Supplementary material for:

Effects of semaglutide on stroke subtypes in type 2 diabetes: *post hoc* analysis of the randomized SUSTAIN 6 & PIONEER 6

W David Strain, M.D.; Ofir Frenkel, M.D.; Martin A. James, F.R.C.P.; Lawrence A. Leiter, M.D.; Søren Rasmussen, Ph.D.; Peter M. Rothwell, F.Med.Sci; Maria Sejersten Ripa, DMSc.; Thomas C. Truelsen TC, DMSc.; Mansoor Husain, M.D.

CONSORT checklist

Section/Topic	Item No	Checklist item	Reported on page No
**Title and abstract**			
	1a	Identification as a randomised trial in the title	Title page
	1b	Structured summary of trial design, methods, results, and conclusions (for specific guidance see CONSORT for abstracts)	Page 1
**Introduction**			
Background and objectives	2a	Scientific background and explanation of rationale	Page 3,4
	2b	Specific objectives or hypotheses	Page 3,4
**Methods**			
Trial design	3a	Description of trial design (such as parallel, factorial) including allocation ratio	Page 4
	3b	Important changes to methods after trial commencement (such as eligibility criteria), with reasons	N/A
Participants	4a	Eligibility criteria for participants	Page 4
	4b	Settings and locations where the data were collected	N/A for this *post hoc* analysis (available in the primary publications)
Interventions	5	The interventions for each group with sufficient details to allow replication, including how and when they were actually administered	N/A for this *post hoc* analysis (available in the primary publications)
Outcomes	6a	Completely defined pre-specified primary and secondary outcome measures, including how and when they were assessed	N/A for this *post hoc* analysis (available in the primary publications); all details related to stroke – the main outcome assessed in this *post hoc* analysis are provided on Page 4
	6b	Any changes to trial outcomes after the trial commenced, with reasons	N/A
Sample size	7a	How sample size was determined	N/A for this *post hoc* analysis (available in the primary publications)
	7b	When applicable, explanation of any interim analyses and stopping guidelines	N/A
Randomisation:			

Sequence generation	8a	Method used to generate the random allocation sequence	N/A for this *post hoc* analysis (available in the primary publications)
	8b	Type of randomisation; details of any restriction (such as blocking and block size)	N/A
Allocation concealment mechanism	9	Mechanism used to implement the random allocation sequence (such as sequentially numbered containers), describing any steps taken to conceal the sequence until interventions were assigned	N/A
Implementation	10	Who generated the random allocation sequence, who enrolled participants, and who assigned participants to interventions	N/A
Blinding	11a	If done, who was blinded after assignment to interventions (for example, participants, care providers, those assessing outcomes) and how	N/A
	11b	If relevant, description of the similarity of interventions	N/A
Statistical methods	12a	Statistical methods used to compare groups for primary and secondary outcomes	Statistical methods for outcomes being investigated in this *post hoc* analysis are on Page 5
	12b	Methods for additional analyses, such as subgroup analyses and adjusted analyses	Page 4–5
**Results**			
Participant flow (a diagram is strongly recommended)	13a	For each group, the numbers of participants who were randomly assigned, received intended treatment, and were analysed for the primary outcome	N/A for this *post hoc* analysis (available in the primary publications)
	13b	For each group, losses and exclusions after randomisation, together with reasons	N/A for this *post hoc* analysis (available in the primary publications)
Recruitment	14a	Dates defining the periods of recruitment and follow-up	N/A for this *post hoc* analysis (available in the primary publications)
	14b	Why the trial ended or was stopped	N/A for this *post hoc* analysis (available in the primary publications)
Baseline data	15	A table showing baseline demographic and clinical characteristics for each group	Table 1, Page 15
Numbers analysed	16	For each group, number of participants (denominator) included in each analysis and whether the analysis was by original assigned groups	Table 1, Pages 5 and 15
Outcomes and estimation	17a	For each primary and secondary outcome, results for each group, and the estimated effect size and its precision (such as 95% confidence interval)	Outcomes in this *post hoc* are on Pages 6-7

	17b	For binary outcomes, presentation of both absolute and relative effect sizes is recommended	N/A
Ancillary analyses	18	Results of any other analyses performed, including subgroup analyses and adjusted analyses, distinguishing pre-specified from exploratory	Page 6-7 (Page 8 of the discussion, stating that this was an exploratory *post hoc* analysis)
Harms	19	All important harms or unintended effects in each group (for specific guidance see CONSORT for harms)	Page 8 and full details are in the primary publications
**Discussion**			
Limitations	20	Trial limitations, addressing sources of potential bias, imprecision, and, if relevant, multiplicity of analyses	Page 8-9
Generalisability	21	Generalisability (external validity, applicability) of the trial findings	Page 8-9
Interpretation	22	Interpretation consistent with results, balancing benefits and harms, and considering other relevant evidence	Pages 7-8
**Other information**			
Registration	23	Registration number and name of trial registry	Pages 1 and 4
Protocol	24	Where the full trial protocol can be accessed, if available	Page 10
Funding	25	Sources of funding and other support (such as supply of drugs), role of funders	Pages 5 and 10

Figure S1: Aalen-Johansen plots for time to first occurrence of any stroke* with subcutaneous semaglutide versus placebo in people with T2D at high CV risk, based on data from the SUSTAIN 6 trial

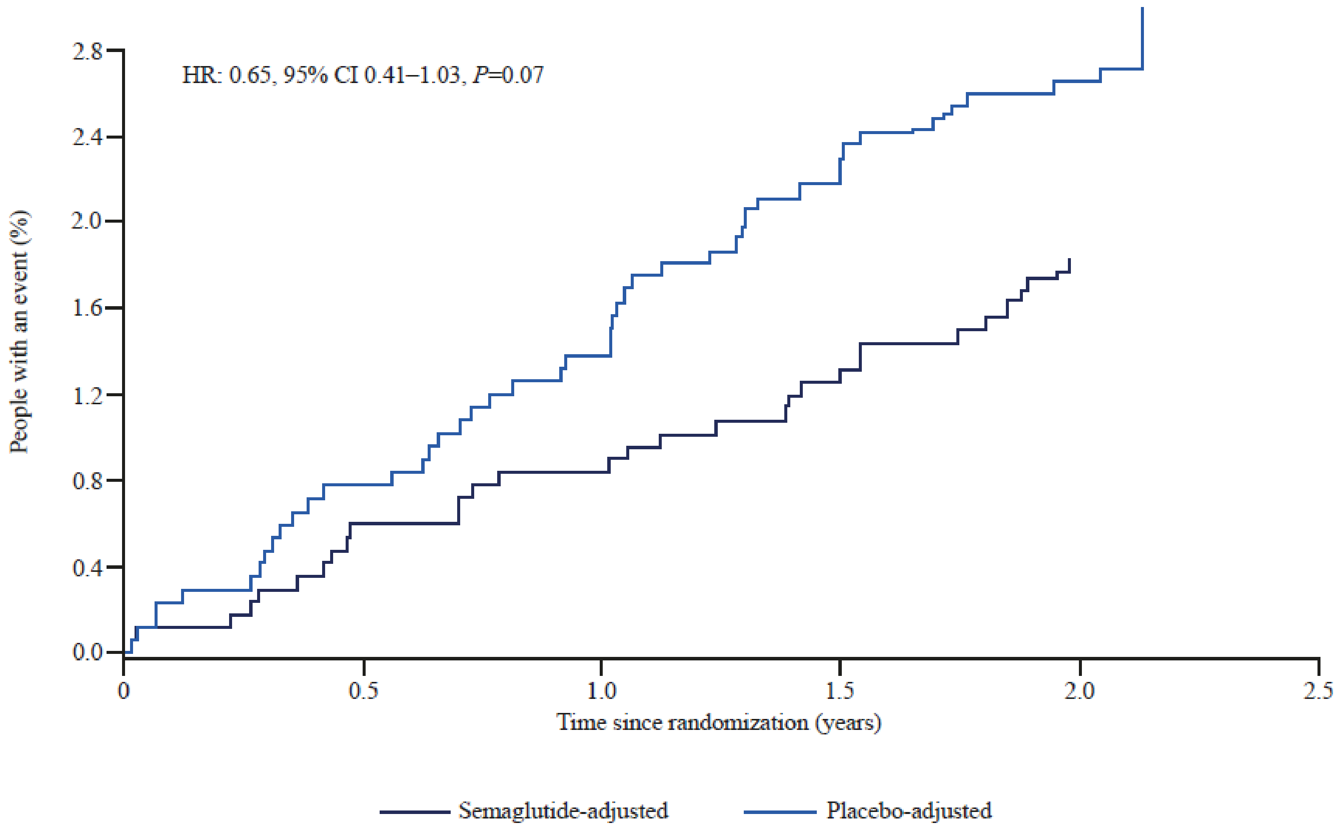

*Included fatal and nonfatal strokes. The cumulative incidence rates for time to first stroke were calculated using Aalen-Johansen method, adjusting for all-cause death as a competing risk. The hazard ratio was estimated from a Cox regression model stratified by trial with treatment as a factor.

CI, confidence interval; CV, cardiovascular; HR, hazard ratio; T2D, type 2 diabetes.

Figure S2: Aalen-Johansen plots for time to first occurrence of any stroke* with oral semaglutide versus placebo in people with T2D at high CV risk, based on data from the PIONEER 6 trial

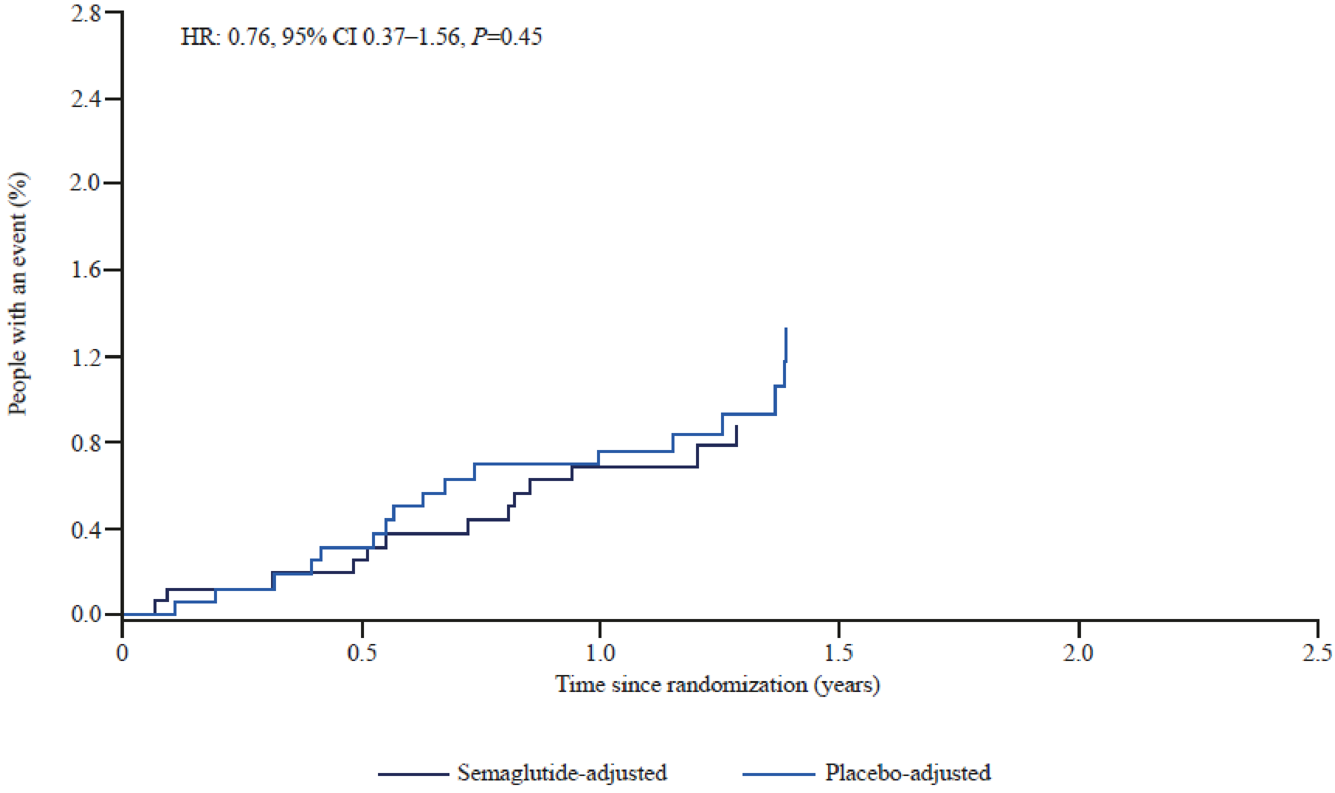

*Included fatal and nonfatal strokes. The cumulative incidence rates for time to first stroke were calculated using Aalen-Johansen method, adjusting for all-cause death as a competing risk. The hazard ratio was estimated from a Cox regression model stratified by trial with treatment as a factor.

CI, confidence interval; CV, cardiovascular; HR, hazard ratio; T2D, type 2 diabetes.
